# Dry Swab-Based Nucleic Acid Extraction vs. Spin Column-Based Nucleic Acid Extraction for COVID-19 RT-PCR Testing: A Comparative Study

**DOI:** 10.1155/2023/6624932

**Published:** 2023-08-23

**Authors:** Mohammed Faraaz Khan, C. Roopa

**Affiliations:** ^1^Department of Microbiology, Kamineni Institute of Medical Sciences, Narketpally, Telangana, India; ^2^Department of Microbiology, SVS Medical College, Mahabubnagar, Telangana, India

## Abstract

Conventional nucleic acid extraction involves usage of spin columns to isolate the RNA, but this is labor intensive. This study compares the spin column method with a dry swab-based method of extraction using a proteinase K buffer and subsequent heat inactivation. A total of 56 subjects were tested for COVID-19 by RT-PCR with probes targeting the E and RdRp genes by collecting two nasopharyngeal and two oropharyngeal swabs and subjecting one set to nucleic acid extraction by spin column and the other set to dry swab-based methods. Out of the 56 samples tested, 27 were positive for VTM-based extraction and 29 were negative. Dry swab-based extraction produced 22 positive results (sensitivity = 81.48%) and 34 negative results. The E gene was detectable in 25 samples by the dry swab method out of 27 samples that tested positive by the VTM-based method (sensitivity = 92.5%). The RdRp gene was detectable in 22 samples by the dry swab method out of 27 samples that tested positive by the VTM-based method (sensitivity = 81.48%). Concordance was 91% with discordance at 9% and a Kappa value of 0.82, indicating almost perfect agreement between the two methods. Our findings indicate that the dry swab method of nucleic acid extraction is a useful alternative to conventional spin column-based extraction with comparable sensitivity and specificity. The trial was registered with the Clinical Trials Registry of India (CTRI) with a CTRI registration number of CTRI/2021/12/038792.

## 1. Introduction

Coronaviruses possess a particular tropism for the respiratory tract as they are very frequent causes of “common cold” like illnesses. Barring a few exceptions, most of the illnesses produced by coronaviruses are mild in nature. However, during the month of December in the year 2019, a novel pneumonia-causing agent was discovered in a cluster of cases in the Chinese city of Wuhan [1]. This agent was found to be a coronavirus which was later named as SARS-CoV-2 due to its genetic similarities with SARS-CoV-1 and the resulting disease was named COVID-19 which has resulted in a global pandemic with unprecedented mortality and morbidity [2].

As of November 2022, there have been a total of 634,522,052 confirmed cases of COVID-19 which have resulted in 6,599,100 deaths, amounting to a mortality rate of 1.04% [3]. In order to rapidly diagnose suspected cases of COVID-19, real-time RT-PCR has been used as the test of choice all over the world. While RT-PCR is a rapid and sensitive means of screening suspected cases of COVID-19, it is not without its technical pitfalls. The performance of the test requires that the viral genetic material be extracted from the intact virions in order to facilitate the amplification process. This process of nucleic acid extraction can be achieved by various means such as using lysis buffers coupled with spin columns to extract the viral RNA or magnetic beads that crush the viral particles to release the RNA. However, these processes can be quite labor-intensive and time-consuming. Moreover, the sudden demand for nucleic acid extraction kits during the pandemic led to a shortage due to a serious imbalance between supply and demand [4]. This necessitated research into alternative methods of nucleic acid extraction to develop other possible methods of extraction so as to ensure a frictionless diagnostic and screening response against the COVID-19 pandemic.

One of the alternatives is the usage of dry nasopharyngeal or oropharyngeal swabs in plain tubes (in the absence of VTM) with pretreatment by a proteinase K TE buffer, followed by heat inactivation, as shown by Kiran et al. [5]. This method presents several advantages. First, it is safer to transport a plain tube containing a dry swab as opposed to a tube filled with VTM as there are no chances of spillage or leakage with a plain tube containing a dry swab [5]. Moreover, this method requires only a single buffer as opposed to the multiple reagents required during nucleic acid extraction by a spin column-based method. The simplicity of the procedure also reduces the time taken to complete nucleic acid extraction as well as the cost involved [5]. The Indian Council of Medical Research (ICMR) has also performed independent validations of the abovementioned extraction method and provided guidelines on the usage of the dry swab-based extraction method to scale up COVID-19 RT-PCR testing in India [6, 7].

This study was conducted to evaluate the efficacy of the dry swab-based method in which a comparative evaluation of both the spin column-based nucleic acid extraction and the dry swab-based nucleic acid extraction was simultaneously performed.

## 2. Materials and Methods

This was a prospective fully paired diagnostic test accuracy study conducted in the department of microbiology at Kamineni Institute of Medical Sciences, Narketpally, from December 2021 to February 2022. A total of 56 patients were enrolled in the study.

### 2.1. Inclusion Criteria

All patients who opted for testing for COVID-19 by RT-PCR and were above the age of 18 years were included in this study.

### 2.2. Exclusion Criteria

Patients who got tested for COVID-19 by other means such as the rapid antigen test and high-resolution computed tomography (HRCT) were excluded from the study. Also excluded patients were all those under the age of 18 years and those who refused to provide consent.

Patient details such as age and gender were documented, with special emphasis on the symptoms and severity of the suspected disease. A total of four swabs were collected from each patient. One nasopharyngeal and one oropharyngeal swab were collected and placed in 10 mL tubes containing 3 mL of VTM. Another set of swabs (one nasopharyngeal and one oropharyngeal) was collected and placed inside plain tubes without VTM. Informed consent was obtained from the patients prior to the collection of the samples. The collected samples were transported to the biosafety level 3 (BSL 3) testing facility of the microbiology department.

The swabs collected in VTM were subjected to nucleic acid extraction using spin columns (PROMEA Therapeutics, Patancheruvu, Telangana), and the extracted RNA was used as the template for RT-PCR.

**Figure 1:**
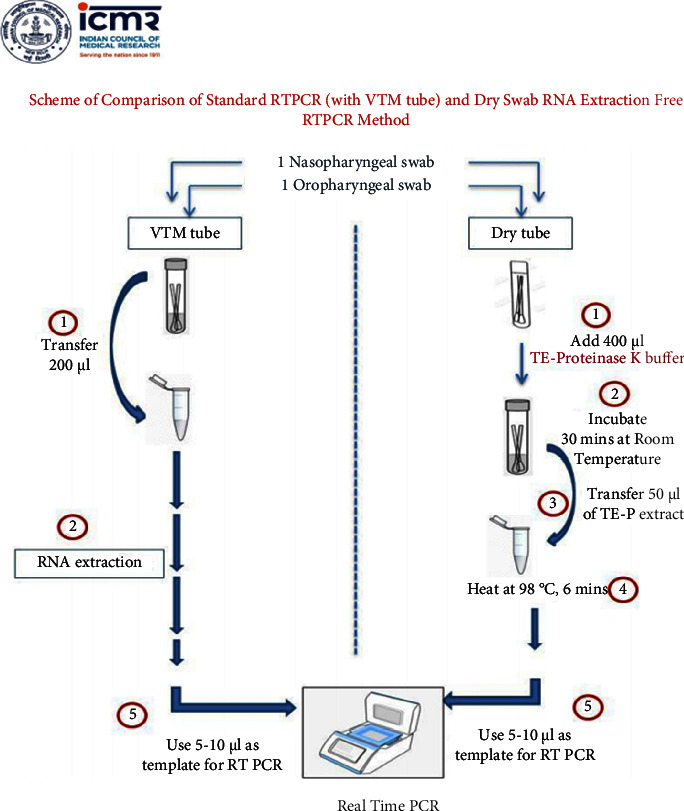
Procedure for the dry swab-based nucleic acid extraction.

The dry swabs collected were treated with 400 *µ*L of a lysis buffer (Meril Diagnostics, Vapi, Gujarat) containing proteinase K and Tris EDTA (proteinase K 2 mg/ml, Tris 7.4 pH 10 mM, and EDTA 0.1 mM), followed by incubation at room temperature for 30 minutes. Later, a 50 *µ*L aliquot was taken into a microfuge tube and heated at 98°C for 6 minutes. This heated aliquot was treated as the PCR template. A schematic for the dry swab-based extraction is given in Figure 1 [7].

After extraction, the RNA from each pair of swabs was subjected to RT-PCR targeting the E and RdRp genes (ProPCR COVID-19 RT-qPCR, PROMEA Therapeutics, Patancheruvu, Telangana) simultaneously inside a 36-well thermal cycler (ROTOR GENEQ 5PLEX, Qiagen). Primers targeting the RNase P gene were provided as internal controls in the RT-PCR kit. The results were read as positive or negative depending on the cycle threshold (*Ct*) values. Any value below 35 with a proper sigmoidal curve of amplification was considered positive, and any value above 35 was considered negative. FAM, HEX, and ROX were the indicator dyes for the RdRp gene, E gene, and the internal control, respectively. A sample was considered positive only if amplification was observed in all three channels. Absence of amplification of the internal control constituted an invalid result, and amplification of only one gene (E gene or RdRp gene) without the other was considered negative. The cycling conditions were as follows: reverse transcription for 15 minutes at 48°C, followed by polymerase activation by holding at 95°C for 3 minutes. This was followed by 45 cycles of PCR, wherein denaturation was carried out at a temperature of 95°C for 10 seconds, with annealing and extension taking place at 55°C for 40 seconds in each cycle. Detection was done at 55°C. The total duration of the RT-PCR run was 110 minutes.

Ethical clearance was obtained from the institutional ethics committee prior to the commencement of the study. All methods used in the study were in accordance with the Declaration of Helsinki. Data compilation and calculation of the mean and standard deviation were done using Microsoft Excel. Statistical analysis was done by using MedCalc Software Ltd. Diagnostic test evaluation calculator (https://www.medcalc.org/calc/diagnostic_test.php (Version 20.118)) and Cohen's Kappa free calculator (https://idostatistics.com/cohen-kappa-free-calculator/). Generation of heat maps was done using heatmapper.ca. The scatter plots were designed using Microsoft Excel.

## 3. Results

Out of the 56 samples tested, 27 tested positive and 29 tested negative by the reference method, whereas the dry swab method of testing resulted in 22 positive and 34 negative results (Table 1). The sensitivity of the dry swab extraction technique was 81.48% (22/27) (95% confidence interval: 61–93) and the specificity was 100% (29/29) (95% confidence interval: 88–100). The positive predictive value was 100%, and the negative predictive value was 85.29% (95% confidence interval: 72–92).

The concordance between the two methods was 91% (95% confidence interval: 80–97) and the discordance was 9%. The Kappa value calculation revealed a value of 0.82 (95% confidence interval: 0.67–0.96). The high degree of concordance and the Kappa value indicate almost perfect agreement between the two methods used.

The E gene was detected in 27 samples by the reference method, whereas the dry swab method yielded positive results for 25 samples (Table 2). The sensitivity of the dry swab method in detecting the E gene was 92.5% (25/27).

The RdRp gene was detected in 27 samples by the reference method, while the dry swab method produced 22 positive results (Table 3). The sensitivity of the dry swab method for detecting the RdRp gene was 81.48% (22/27).

Fever was the most common symptom (46.4%) noted in the study population (26/56), whereas anosmia (3.5%) was the rarest (2/56). The symptoms were not mutually exclusive, with many patients exhibiting multiple symptoms. A fair number of patients were asymptomatic (33.9%) as well (19/56) (Table 4).

**Figure 2:**
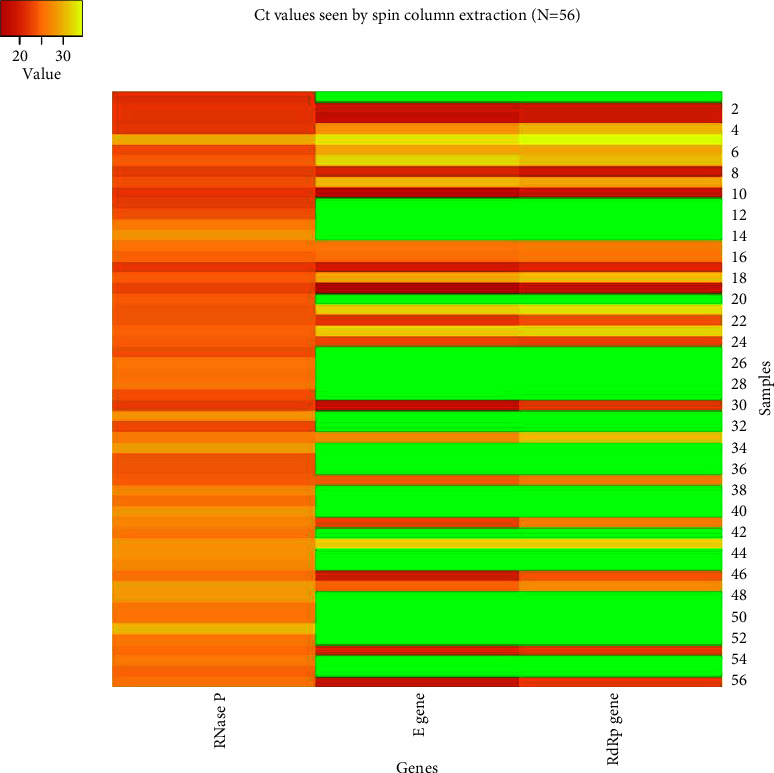
Heat map demonstrating the *Ct* values for RNase P (internal control), E gene, and RdRp genes in samples tested by the spin column-based extraction method. The green color indicates no *Ct* values for that particular gene.

**Figure 3:**
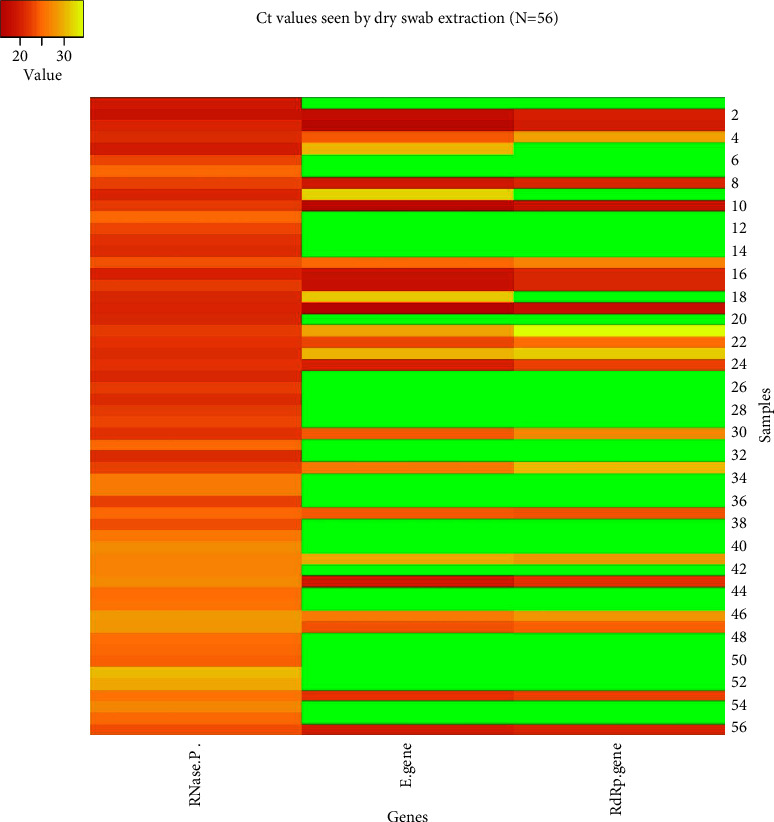
Heat map demonstrating the *Ct* values for RNase P (internal control), E gene, and RdRp genes in samples tested by the dry swab-based extraction method. The green color indicates no *Ct* values for that particular gene.

**Figure 4:**
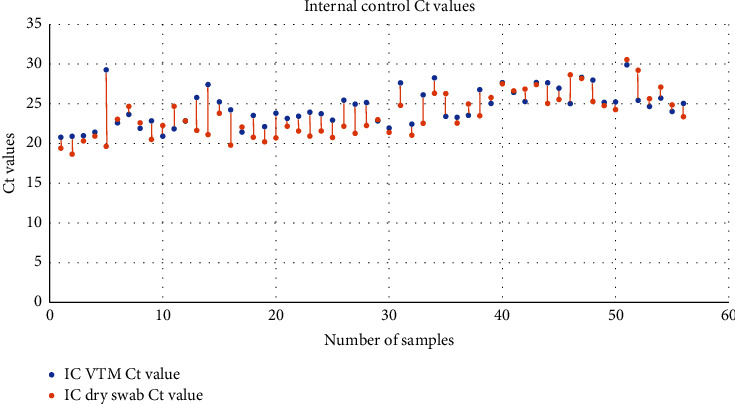
Scatter plot showing the *Ct* values for internal controls extracted by the spin column method and dry swab method.

**Figure 5:**
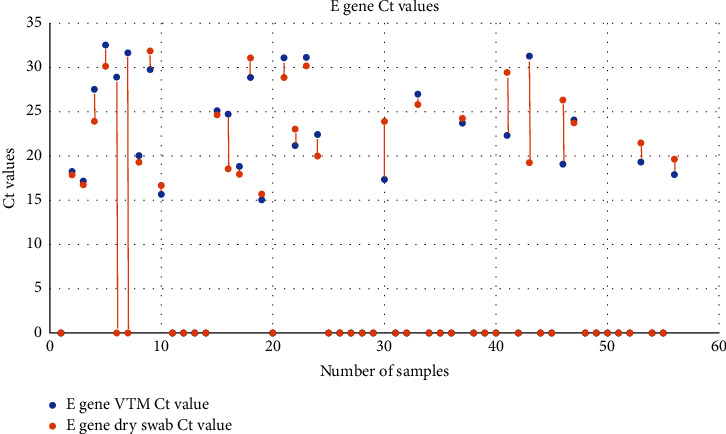
Scatter plot showing the *Ct* values for the E gene extracted by the spin column and dry swab-based method.

**Figure 6:**
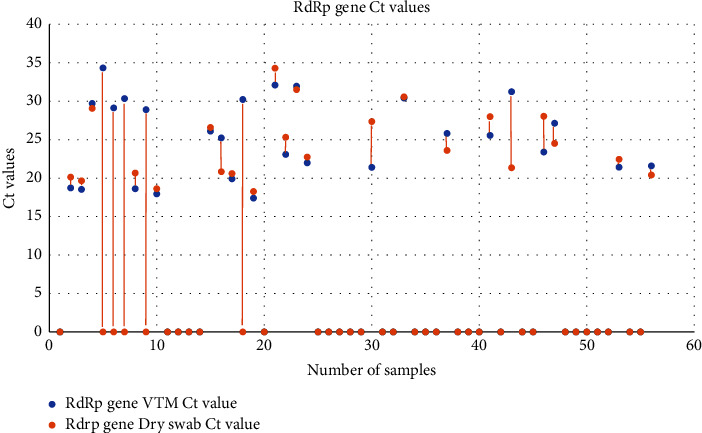
Scatter plot showing the *Ct* values for the RdRp gene extracted by the spin column and dry swab-based methods.

The *Ct* (cycle threshold) values obtained in both the methods were compared as well. The mean *Ct* values obtained for each target by the spin column method were 24.57 ± 2.28, 23.77 ± 5.45, and 25.27 ± 5.01 for the RNase P (internal control), E gene, and RdRp genes, respectively. In case of dry swab-based extraction, the mean *Ct* values were 23.49 ± 2.74, 23.21 ± 4.90, and 24.30 ± 4.44 for the RNase P (internal control), E gene, and RdRp genes, respectively (Figures 2–6).

## 4. Discussion

The COVID-19 pandemic has resulted in not just mass morbidity and mortality worldwide but has also produced an immense strain on the diagnostic services, resulting in shortages of essential diagnostic reagents worldwide. In lieu of these challenges, several authors have attempted to circumvent the cumbersome steps of nucleic acid extraction by various means.

Srivatsan et al. conducted a study using two self-collected nasal swabs from each subject to assess the efficacy of transport medium-free extraction using proteinase K and a TE buffer, followed by heating the samples at 37° Celsius for 15 minutes and 95° Celsius for 15 minutes [8]. One swab was transported in an empty tube and was subjected to the protocol as mentioned above, whereas the other swab was transported in a tube containing universal transport medium (UTM) and treated to nucleic acid extraction by magnetic beads [8]. The RT-PCR was done targeting the S and ORF1ab genes [8]. The findings were encouraging, with a reported sensitivity of 100% and a specificity of 99.4% [8]. While the present study shared a similar specificity of 100%, the sensitivity was lower at 81.48%. However, the study was biased in the sense that it recruited only patients who were known cases of lab-diagnosed COVID-19 [8].

A similar study was performed by Nique et al., wherein 78 samples were collected from suspected COVID-19 patients [9]. The nasopharyngeal swabs were collected in UTM and transported to the laboratory following which they were subjected to RNA extraction by a commercial kit and an in-house proteinase K extraction protocol simultaneously [9]. Treatment with proteinase K was followed by incubation of the UTM at 56°Celsius for 10 minutes following which a thermal shock was performed by heating the sample at 98° Celsius for 5 minutes and subsequently cooling it at 4° Celsius for 2 minutes [9]. The RT-PCR protocol used primers targeting the RdRp gene (as a marker of SARS-CoV-2) and glyceraldehyde phosphate dehydrogenase (GAPDH) as an internal control [9]. Nique et al. reported 100% sensitivity and specificity with 100% concordance in their study, as opposed to the present sensitivity of 81.48% and concordance of 91%, although the specificity in the present study was 100% as well [9].

In contrast to the aforementioned studies, the extraction protocol used in the present study did not utilize any transport medium. The protocol was in fact in accordance with the extraction process proposed by Kiran et al. [5] In their study, Kiran et al. collected two nasopharyngeal swabs from each patient of which one was collected in viral transport medium (VTM) and subjected to kit-based RNA extraction [5]. The other swab was collected in an empty tube and treated with 400 *µ*L of a proteinase K TE buffer for 30 minutes, followed by heat inactivation at 98° Celsius for 6 minutes [5]. All the extracted samples were subjected to RT-PCR targeting the E and RdRp genes. Out of the 40 samples tested, 23 were positive by the index method, whereas 19 were positive by the dry swab method which gave rise to a sensitivity of 82.6% which was very similar to the present sensitivity of 81.48%. The specificity, however, was only 82.35% which was inferior to the specificity of 100% obtained in the present study [5]. The concordance between the two methods was 91% in the present study which was superior to the concordance of 82.5% noted by Kiran et al. [5].

Jayaprakasam et al. from the Indian Council of Medical Research (ICMR) conducted a large-scale two-site study to validate the extraction protocol as devised by Kiran et al. [5, 6] 1138 samples were collected in total from both the study sites. One pair of swabs (one nasopharyngeal and one oropharyngeal) was subjected to kit-based extraction after collection in VTM, whereas the other pair of swabs was subjected to the dry swab extraction protocol as outlined by Kiran et al. [5, 6]. The genes targeted by the RT-PCR primers were the E, N, and RdRp genes. 128 samples were reported to be positive by the reference method, whereas 101 specimens returned a positive result by the dry swab method (sensitivity = 78.9%) [6]. While the sensitivity in Jayaprakasam et al.'s study was only marginally lower than the sensitivity noted in the present study (81.48%), the specificity noted by Jayaprakasam et al. (99%) was similar to what was obtained in the present study (100%) [6]. Moreover, Jayaprakasam et al. noted a very high concordance (96.8%) between the two methods which was higher than the concordance noted in the present study (91%) [6]. The Kappa values were highly similar (0.83 in Jayaprakasam et al. and 0.82 in the present study), indicating almost perfect agreement between the two methods, which was a finding shared in both the studies [6]. The findings of this study enabled ICMR to validate the dry swab-based extraction method for nationwide use [7].

Dhakad et al. performed a study on 211 patients [10]. The investigators collected two sets of nasopharyngeal and oropharyngeal swabs, transporting one set in a VTM and subjecting it to kit-based extraction, while the other set was transported in a dry tube and subjected to dry swab-based extraction as prescribed by ICMR [7]. Primers targeting the E and ORF genes were used in the RT-PCR process. The authors reported 66 positive results by the reference method; however, only 26 of these samples were tested by the dry swab method as well [10]. Both the sensitivity (81.48%) and specificity (100%) in the present study were superior to those of Dhakad et al. (sensitivity = 39.39% and specificity = 85.71%) [10]. This led to a concordance of only 49.59% which was inferior to the 91% obtained in the present study [10].

A study by Michel et al. compared a kit-independent method of detection of SARS-CoV-2 RNA called COVID-quick-DET, with standard kit-based RNA isolation techniques [11]. The collected dry swabs were resuspended in 1 mL of normal saline with concurrent vortexing of the specimens for 5 seconds [11]. This was followed by treatment with 3 *µ*L of proteinase K (20 mg/mL) to around 80–100 *µ*L of the samples [11]. The samples were subsequently subjected to a spin at 8,000 rpm for 2 to 5 seconds [11]. Subsequently, all samples were subjected to heat inactivation at 56°C for three minutes followed by 95°C for three minutes as well [11]. PCR primers targeting the E gene and ORF1b-nsp14 were used [11]. The authors reported a sensitivity of 94.64% in the 56 samples tested [11]. This was higher than the sensitivity of 81.48% obtained in the present study. The specificity shown by Michel et al. was 100% which was similar to the specificity of 100% obtained in the present study [11]. However, the disadvantage of the study by Michel et al. lies in the fact that samples with known results were used for comparison [11]. The 56 samples tested by Michel et al. were from known lab-confirmed cases of COVID-19 of which 3 were negative by the COVID-quick-DET method to give a sensitivity of 94.64%, while another 13 known lab-confirmed COVID-19-negative specimens were tested by the COVID-quick-DET method to give a specificity of 100% [11].

Genoud et al. performed a study to evaluate a protocol combining proteinase K and heat treatment for the extraction of RNA from nasopharyngeal swabs collected from suspected COVID-19 patients in tubes of normal saline [12]. Nasopharyngeal swabs were collected in around 2 to 5 mL of normal saline in tubes for kit-based extraction and proteinase K-based extraction [12]. The samples were vortexed, and 90 *µ*L of the nasopharyngeal samples were added to 0.2 mL PCR tubes, to which 10 *µ*L of a 10 mg/mL proteinase K solution had been previously added [12]. The tubes were incubated at 55°C for 15 minutes, followed by heat inactivation at 98°C for 5 minutes [12]. Multiple kits targeting multiple regions of the viral genome were used in the study of which one of the kits included primers targeting the N, E, and RdRp genes [12]. A total of 94 random samples were tested by this kit, producing a sensitivity of 92% and a specificity of 96% [12]. While the sensitivity was noted to be higher as compared to the sensitivity of 81.48% obtained in the present study, this was probably due to the inclusion of the N gene in the detection kit [12]. The specificity was slightly lower than the specificity of 100% obtained in the present study.

Chu et al. conducted a study with 50 specimens to evaluate the efficiency of a protocol using proteinase K and heat treatment for the extraction of RNA from SARS-CoV-2 [4]. Of the 50 specimens, 25 were nasopharyngeal swabs collected in VTM [4]. The proteinase K solution was added to the nasopharyngeal swabs in a ratio of 1 : 5, followed by incubation at 56°C for 15 minutes and subsequently a final heat treatment at 98°C for 5 minutes [4]. Primers targeting the RdRp genes and helicase genes were used in the RT-PCR mix [4]. 21 of the 25 samples tested positive (sensitivity = 84%), and 4 tested negative [4]. The sensitivity was slightly higher than the sensitivity (81.48%) obtained in the present study.

Bruce et al. performed an assessment of a direct RT-qPCR method with heating of 3 *µ*L of the nasopharyngeal swabs containing diluent at 95°C for 10 minutes [13]. A total of 150 samples were tested using this protocol, wherein all 150 of these specimens belonged to individuals who had tested positive for COVID-19 when tested with RT-qPCR at a separate site [13]. Primers targeting the N1 and N2 genes were used to perform the RT-q PCR reaction [13]. The heat inactivation protocol detected the RNA in 138 of the 150 specimens, resulting in a sensitivity of 92% [13]. This was higher as compared to the sensitivity obtained in the present study (81.48%).

One of the important findings in our study was the reduced sensitivity of the dry swab method in the detection of the RdRp gene (81.48%) as compared to the E gene (92.5%). While the dry swab method of extraction could be considered as a comparable alternative to spin column-based extraction for the detection of the E gene, the same cannot be said wholeheartedly for the RdRp gene. This problem has been encountered in other studies as well. Genoud et al. reported a higher sensitivity with respect to the detection of N1 and N2 genes as opposed to the RdRp gene using their proteinase K and heat inactivation-based extraction protocol [12]. Smyrlaki et al. reported similar findings [14]. Using a heat inactivation protocol followed by RT-q PCR (which was labelled as hid-RT-PCR), they showed that the Ct values obtained by the various genes were considerably different [14]. They observed that the Ct values obtained from amplification of the N1 gene were the lowest, followed by those obtained by the RdRp gene and lastly by the E gene [14]. A possible explanation is the heat-induced fragmentation of the genes during the heating process carried out during the heat-based extraction procedures [14]. The length of the N1 gene is around 72 kbp in contrast to the RdRp gene which is around 81 kbp [14]. This does not assume much significance when solid phases such as spin columns and magnetic beads are used for nucleic acid extraction. However, when extraction procedures involving heat are applied, there lies a potential danger of fragmentation of the genomic segments. Shorter amplicons such as the N1 genes are comparatively unaffected as opposed to the lengthier amplicons such as the RdRp genes which undergo denaturation. This leaves a lower amount of target genes in the reaction mixture, resulting in a failure of amplification of the target genes and resultant false-negative results. While the detection of the E gene was higher in the present study in contrast to the aforementioned studies, the detection of the RdRp gene was in line with the lower sensitivity and higher *Ct* values as reported by Smyrlaki et al. and Genoud et al. [12, 14]. As such, selection of the right amplification targets becomes imperative when a heat-based extraction method is applied as opposed to extraction carried out with other nonthermal means [14].

Another observation that merits discussion is with regards to the lower sensitivity of the dry swab-based method of extraction in the detection of the RdRp gene. Amplification of the RdRp gene was seen only in 22 of the patient samples, while the gene was detected in 27 samples by the spin column-based extraction method. On the contrary, the E gene was detected in 25 of the samples by the dry swab method. This points towards a better sensitivity for the detection of the E gene as opposed to the RdRp gene. The causes of this discrepancy could be multiple. One of the potential reasons for the lower sensitivity is the aforementioned thermal fragmentation of the RdRp gene. Another important point is with regards to the relative concentration of the target RdRp gene in the samples that tested negative. As the RT-PCR performed in this study was qualitative, a rough estimate of the target gene concentration in the sample could be acquired by noting the *Ct* value of the amplicon. The mean *Ct* value of the five samples in which the RdRp gene was undetectable by the dry swab-based extraction was 30.59 ± 1.49. As a *Ct* value of 35 was taken as the cutoff in this study, a mean *Ct* value of 30.59 ± 1.49 is more towards the upper limit of detection, indicating that the concentration of the RdRp gene in these samples was probably lower to begin with. Moreover, these samples were from asymptomatic individuals who were recovering from the infection. Their reason for getting tested was to ensure a negative result prior to their return to their respective workplaces after the completion of their 14-day isolation. All this points to the possibility that the detected nucleic acid was likely a low-level noninfective shedding from these patients. As far as symptomatic cases go, the dry swab-based method was successful in detecting the infection in all of them.

As far as the discrepancy between the sensitivity of the E gene and RdRp gene detection goes, it can possibly be explained by looking into the inherent nature of the virus. Subgenomic RNA corresponding to the E gene is expressed much more abundantly than the nonstructural protein 12 gene that codes for the RNA-dependent RNA polymerase [15]. The maximum expression is noted for the N gene subgenomic RNA while the expression of the E gene subgenomic RNA is much inferior with the RdRp gene having the lowest amount of expression among the three [15]. This genetic subtlety is most likely the reason as to why the detection sensitivity for the E gene is higher than that of the RdRp gene in the present study, as the target E genes are much more abundantly present in the clinical samples as opposed to the fewer molecules of the RdRp genes. Further studies with multiple primer sets such as those using the E gene, RdRp gene, N gene, and ORF1ab are recommended to determine the optimal primer probe set to use in this dry swab-based extraction technique, as the sensitivities may vary with different genetic targets due to several factors.

## 5. Conclusion

The dry swab-based extraction method has been demonstrated to be a useful tool in the fight against the COVID-19 pandemic. The findings in this study show that the aforementioned method of extraction has a high degree of concordance with the solid-phase spin column-based extraction method. The efficiency of the dry swab-based method was comparable to the conventional spin column-based method for the E gene but not for the RdRp gene. While further studies to assess the usefulness of the dry swab-based extraction are recommended with other genome targets such as the N1 gene, N2 gene, and ORF1ab gene, the overall agreement between the two nucleic acid extraction methods was almost perfect. In conclusion, the dry swab-based method of nucleic acid extraction is a useful alternative to the spin column-based method of extraction with a high degree of specificity albeit a slightly lowered sensitivity.

## Figures and Tables

**Table 1:** Comparison of RT-PCR results between the two methods of extraction (*N* = 56).

Results	Spin column extraction	Dry swab extraction
Positive	27	22
Negative	29	34
Total	56	56

**Table 2:** Comparison of sensitivity for the detection of the E gene between the two extraction methods (*N* = 56).

Results	Spin column extraction	Dry swab extraction
Positive	27	25
Negative	29	31
Total	56	56

**Table 3:** Comparison of sensitivity for the detection of the RdRp gene between the two extraction methods (*N* = 56).

Results	Spin column extraction	Dry swab extraction
Positive	27	22
Negative	29	34
Total	56	56

**Table 4:** Spectrum of symptoms among the recruited patients (*N* = 56).

Symptoms	Frequency
Fever	26 (46.4%)
Sore throat	22 (39.2%)
Cough	19 (33.9%)
Body ache	17 (30.3%)
Diarrhoea	5 (8.9%)
Ageusia	3 (5.3%)
Dyspnea	3 (5.3%)
Anosmia	2 (3.5%)
Asymptomatic	19 (33.9%)
Total	56

## Data Availability

The data presented in this study are available upon reasonable request from the corresponding author. The data are not shared publicly due to ethical reasons.
